# 473. Trends among hospitalized pregnant patients with laboratory-confirmed SARS-CoV-2 — COVID-NET, 14 U.S. States, January 2021–January 2023

**DOI:** 10.1093/ofid/ofad500.543

**Published:** 2023-11-27

**Authors:** Fiona P Havers, Michael Whitaker, Jennifer Milucky, Monica E Patton, Bhoomija Chatwani, Shua Chai, Isaac Armistead, James Meek, Kyle P Openo, Andy Weigel, Patricia A Ryan, Val Tellez Nunez, Erica Bye, Dominic Rudin, Kerianne Engesser, Sophrena Bushey, Nancy E Moran, Melissa Sutton, H Keipp Talbot, Kirsten Olsen, Christopher Taylor

**Affiliations:** CDC, Atlanta, Georgia; CDC, Atlanta, Georgia; Centers for Disease Control and Prevention, Atlanta, Georgia; CDC, Atlanta, Georgia; Eagle Health Analytics LLC, San Antonio, Texas; Centers for Disease Control and Prevention, Atlanta, Georgia, Atlanta, Georgia; California Department of Public Health, Oakland, CA; Colorado Department of Public Health and Environment, Denver, Colorado; Connecticut Emerging Infections Program, Yale School of Public Health, New Haven, Connecticut; Georgia Emerging Infections Program and Atlanta VA Medical Center, Decatur, GA; Iowa Department of Public Health, Des Moines, Iowa; Maryland Department of Health, Baltimore, Maryland; Michigan Department of Health and Human Services, Lansing, Michigan; Minnesota Department of Health, Saint Paul, Minnesota; University of New Mexico, Albuquerque, New Mexico; New York State Department of Health, Albany, New York; University of Rochester School of Medicine and Dentistry, Rochester, New York; Ohio Dept of Health, Columbus, Ohio; Oregon Health Authority, Portland, Oregon; Vanderbilt University Medical Center, Nashville, Tennessee; Local Health Department, Salt Lake City, Utah; CDC, Atlanta, Georgia

## Abstract

**Background:**

Pregnant persons are at increased risk for adverse outcomes from COVID-19 infection. We describe trends and clinical outcomes among pregnant patients hospitalized with a positive SARS-CoV-2 test.

**Methods:**

From January 2021–January 2023, hospitalized pregnant patients aged 15–49 years with a positive screening or clinician-directed SARS-CoV-2 test ≤ 14 days prior to or during hospitalization were identified from > 300 hospitals across 99 counties in 14 states in the COVID-19-Associated Hospitalization Surveillance Network (COVID-NET). Trained staff conducted chart abstractions on a representative sample of patients. We examined trends in the proportion of pregnant patients with COVID-19 respiratory symptoms and, among those with respiratory symptoms, trends in demographics and clinical outcomes from January–November 2021 (pre-Omicron), December 2021–June 2022 (early Omicron) and July 2022–January 2023 (later Omicron). Percentages presented were weighted to account for the probability of selection for sampled cases.

**Results:**

Out of 1,637 hospitalized pregnant patients with SARS-CoV-2 infection, respiratory symptoms were recorded for 359 (21.9%). The proportion without respiratory symptoms increased from 73.4% (pre-Omicron) to 82.1% (later Omicron). Over the study period, among those with respiratory symptoms, the proportion with ≥1 underlying medical condition increased from 32.7% to 61.5% (**Table 1**), while the proportion requiring intensive care unit admission or mechanical ventilation decreased from 17.3% to 7.0% and from 7.2% to 0.0%, respectively (**Table 2**). During the later Omicron period, among patients with respiratory symptoms, 37.8% were unvaccinated, 35.7% had received a primary vaccination series only and 18.7% had received ≥1 booster doses **(Table 2)**; a higher proportion (29.3%) of asymptomatic patients had received ≥1 boosters in the same period (**Figure**).

**Table 1.**
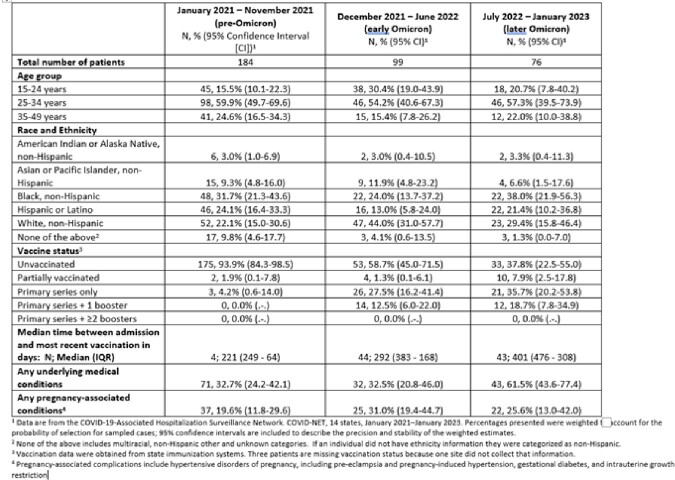
Characteristics of hospitalized pregnant patients (aged 15–49 years) with respiratory symptoms and a positive SARS-CoV-2 test, by pandemic period—COVID-NET, 14 U.S. States, January 2021–January 2023

**Table 2.**
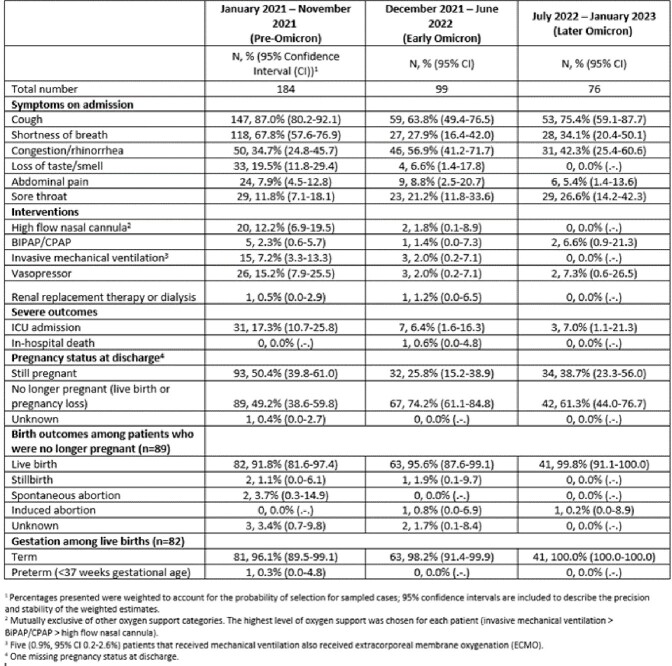
Clinical characteristics among hospitalized pregnant patients (15–49 years) with respiratory symptoms and a positive SARS-CoV-2 test—COVID-NET, 14 U.S. States, January 2021–January 2023

**Conclusion:**

The proportion of symptomatic pregnant patients hospitalized with COVID-19 who had severe clinical outcomes decreased over time, although adverse clinical outcomes continue to occur. Most symptomatic hospitalized pregnant patients were not up to date with COVID-19 vaccination during a period when COVID-19 booster doses were available.

**Disclosures:**

**All Authors**: No reported disclosures

